# Biopotential of *Bersama abyssinica* Fresen Stem Bark Extracts: UHPLC Profiles, Antioxidant, Enzyme Inhibitory, and Antiproliferative Propensities

**DOI:** 10.3390/antiox9020163

**Published:** 2020-02-17

**Authors:** Kouadio Ibrahime Sinan, Annalisa Chiavaroli, Giustino Orlando, Kouadio Bene, Gokhan Zengin, Zoltán Cziáky, József Jekő, Mohamad Fawzi Mahomoodally, Marie Carene Nancy Picot-Allain, Luigi Menghini, Lucia Recinella, Luigi Brunetti, Sheila Leone, Maria Chiara Ciferri, Simonetta Di Simone, Claudio Ferrante

**Affiliations:** 1 Department of Biology, Science Faculty, Selcuk Universtiy, Campus, Konya, 42130 Konya, Turkey; sinankouadio@gmail.com; 2 Department of Pharmacy, “G. d’Annunzio” University Chieti-Pescara, 66100 Chieti, Italy; annalisa.chiavaroli@unich.it (A.C.); luigi.menghini@unich.it (L.M.); lucia.recinella@unich.it (L.R.); luigi.brunetti@unich.it (L.B.); sheila.leone@unich.it (S.L.); disimonesimonetta@gmail.com (S.D.S.); claudio.ferrante@unich.it (C.F.); 3 Laboratoire de Botanique et Phytothérapie, Unité de Formation et de Recherche Sciences de la Nature, 02 BP 801 Abidjan 02, Université Nangui Abrogoua, Abidjan 02, Cote D’Ivoire; kouadio777@gmail.com; 4 Agricultural and Molecular Research and Service Institute, University of Nyíregyháza, 4400 Nyíregyháza, Hungary; cziaky.zoltan@nye.hu (Z.C.); jjozsi@gmail.com (J.J.); 5 Institute of Research and Development, Duy Tan University, Da Nang 550000, Vietnam; 6 Department of Health Sciences, Faculty of Science, University of Mauritius, Réduit 230, Mauritius; picotcarene@yahoo.com

**Keywords:** Africa, acetylcholinesterase, Mangiferin, kynurenic acid, serotonin, colon cancer

## Abstract

In this study, ethyl acetate, methanol, and water extracts of *Bersama abyssinica* (Melianthaceae) stem bark were screened for enzyme inhibitory and antioxidant properties. The water extract possessed the highest concentration of phenols (230.83 mg gallic acid equivalent/g extract), while the methanol extract was rich in flavonoids (75.82 mg rutin equivalent/g extract), and the ethyl acetate extract possessed the highest amount of saponins (97.37 mg quillaja equivalent/g). The aim of this study was to investigate the antiproliferative effects against the human colon cancer HCT116 cell line challenged with serotonin (5-HT) as a stimulating-proliferation factor. The level of HCT116 cell-deriving pool of kynurenic acid (KA) was also assessed. The UHPLC results confirmed the presence of 58, 68, and 63 compounds in the ethyl acetate, methanol, and water extracts, respectively. Mangiferin, vitexin and its isomer isovitexin were tentatively identified in all extracts and KA (*m*/*z* 190.05042 [M−H]^+^) was also tentatively identified in the methanol and water extracts. The methanol extract (1464.08 mg Trolox equivalent [TE]/g extract) showed the highest activity in the CUPRAC assay, whereas the water extract (1063.70 mg TE/g extract) showed the highest activity with the FRAP technique. The ethyl acetate extract was the most active acetylcholinesterase (4.43 mg galantamine equivalent/g extract) and α-glucosidase (mmol acarbose equivalent /g extract) inhibitor. The water extract was able to inhibit 5-HT-stimulated viability of HCT116 cells, and blunt 5-HT-induced reduction of cell-deriving KA. The scientific data generated in this study provide baseline data regarding the biological properties of *B. abyssinica* stem bark, highlighting its potential use for the development of new pharmaceutic and cosmetic agents.

## 1. Introduction

Since ancient times, populations around the globe have relied on plants for food and medicinal purposes. Today, medicinal plants remain the most accessible source of therapeutics for the management of multiple ailments. Over the past decades, researchers from academic institutions to pharmaceutical industries have shown significant interest in natural products including plants and this shift has been driven by increased public interest in plants. Compounds from plants can be used as templates for the development of novel pharmaceutical agents, as well as in the form of botanical products or supplements referred to as complementary and alternative medicine for the prevention and management of diseases. One of the major challenges of scientists is to sustain alleged traditional therapeutic properties of medicinal plants and to rationalize proper dosage for safe use. In this context, research plays a pivotal role in the quest for novel candidates from plants. However, there is still a lack of scientific attention on bioactive compounds from many plants (especially wild plants).

*B. abyssinica* (Melianthaceae) occurring across regions of Sub Saharan Africa, has been used in traditional medicine for the management of multiple ailments, such as colic, diarrhea, dysentery, intestinal worms, rabies, gonorrhea, syphilis, malaria, diabetes, lumbago, fever, debility, hemorrhoids, epilepsy, cancer, rheumatism, menstrual pain, leprosy, impotence, snake bites, and liver disease [1,2,3]. *B. abyssinica* was reported to be traditionally used for treating patients with malignancies [4]. Later studies revealed that bufadienolides isolated from the alcoholic extract of *B. abyssinica* showed cytotoxic activity [5]. Hellebrigenin 3-acetate (I) and hellebrigenin 3,5-diacetate (II), isolated from *B. abyssinica* alcoholic stem bark extracts, showed cytotoxic activity against human nasopharynx cancer cells [6]. Another study reported the antimicrobial activity of the ethyl acetate extract of *B. abyssinica* leaves containing picolinyl hydrazide [7]. 

Currently, there is a lack of scientific literature on the possible enzyme inhibitory activity of *B. abyssinica* stem bark, and therefore this presents a promising avenue for bioprospection. In this study, the enzyme inhibitory properties and antioxidant activity of the ethyl acetate, methanol, and water extracts of *B. abyssinica* stem bark were determined. Considering the traditional antitumoral use [4], antiproliferative effects were investigated against the human colon cancer HCT116 cell line challenged with serotonin (5-HT), a well-known central neurotransmitter, that acts in the periphery and, particularly in the gut, as a proinflammatory and mitogen factor [8,9,10]. It is expected that data generated from this study could provide further scientific information on *B. abyssinica* for future works. 

## 2. Materials and Methods

### 2.1. Plant Material and Preparation of Extracts

The plant samples were collected from the Gontougo region (Nioumassi, location of the area is between 7°0’00” and 9°0’00” North Latitude and 4°5’00” and 5°5’00” West Longitude) of the Ivory Coast, in 2018 (summer season), and were identified by the botanist, Dr. Kouadio Bene, from the Laboratoire de Botanique et Phytothérapie, Université Nangui Abrogoua, Abidjan, Côte d’Ivoire. The stem barks were collected from ten plants in the same population. The stem barks were dried at room temperature for ten days. A laboratory mill was used to grind the samples.

The extraction procedure was conducted following maceration (for ethyl acetate and methanol) and infusion (for water) methods. Briefly, for maceration, 5 g powdered plant sample was stirred with solvents (100 mL) overnight at room temperature. Then, the solvents were evaporated using a rotary evaporator. For water extracts, 5 g powdered plant sample in boiled water (100 mL) was allowed to stand for 20 min. Then, the aqueous extract was lyophilized and all extracts were kept at +4 °C until use.

### 2.2. Profile of Bioactive Compounds

The total bioactive compounds were determined colorimetrically, as described previously [11,12,13]. The results were expressed as mg of standard compounds (gallic acid for phenolic, rutin for flavonoids, caffeic acid for total phenolic acid, catechin for total flavanol and tannins, and quillaja for saponins) per g of dried plant extract. Bioactive profile of the *B. abyssinica* extracts was determined using a Dionex Ultimate 3000RS UHPLC instrument. All analytical and chromatographic details are given in Supplementary Materials and some earlier papers were used to identify some compounds [14,15]. The quantitative determination of rutin and gallic was also performed through independent high-performance liquid chromatography (HPLC) coupled to fluorometric detection. The experimental conditions were selected according to our previous published paper [16]. The levels of gallic acid and rutin were expressed as mg/g dry extract. 

### 2.3. Determination of Antioxidant and Enzyme Inhibitory Effects

For antioxidant capacity, we used different test systems, including radical quenching, reducing power, phosphomolybdenum, and ferrous ion chelating. Details of the methods used were described in our earlier paper [17]. Results were expressed as the standard equivalent of trolox and EDTA for ferrous ion chelating [18]. For enzyme inhibitory effects, key enzymes for global health problems were selected, namely α-amylase and α-glucosidase, acetylcholinesterase (AChE), butyrylcholinesterase (BChE), and tyrosinase. Similar to antioxidant assays, results for enzyme inhibition were expressed as the standard equivalent of acarbose for α-amylase and α-glucosidase; galatamine for AChE and BChE, and kojic acid for tyrosinase [18]. Data were the means ± SD of three replications. Then, one-way ANOVA (Tukey’s assay) was performed under Xlstat 2017 software (*p* < 0.05 considered to be statistically significant) for determining differences in the extracts.

### 2.4. Artemia Salina Lethality Test

In *Artemia salina* lethality bioassay, brine shrimp larvae were incubated for 24 h with *B. abyssinica* extracts (0.1 to 20 mg/mL) dissolved in incubation medium (artificial sea water). The detailed protocol is described in our previous article [19].

### 2.5. Human Colon Cancer HCT116 Cell Culture

HCT116 cell line (ATCC^®^ CCL-247™, ATCC Company, Manassas, Virginia, USA) was cultured in DMEM (Euroclone) supplemented with 10% (*v*/*v*) heat-inactivated fetal bovine serum and 1.2% (*v*/*v*) penicillin G/streptomycin in 75 cm^2^ tissue culture flask (*n* = 5 individual culture flasks for each condition), as previously reported [19]. 

To assess the basal cytotoxicity of *B. abyssinica* extract, a viability test was performed on 96 microwell plates, using 3-(4,5-dimethylthiazol-2-yl)-2,5-diphenyltetrazolium bromide (MTT) test. Cells were incubated with extracts (0.1 mg/mL) for 24 h. An aliquot of 10 μL of MTT (5 mg/mL) was added to each well and incubated for 3 h. In the same condition, the kynurenic acid (KA) extracellular level was determined through HPLC-fluorimeter, as recently described [16].

Finally, we tested extracts on HCT116 cell spontaneous migration, in the wound healing experimental paradigm. The details about MTT and the wound healing test are described in our previous paper [19]. 

### 2.6. Statistical Analysis

Results of in vitro studies (pharmacological assays) were expressed as means ± standard deviation (SD) of three experiments performed in triplicate. Statistical analysis was determined through analysis of variance (ANOVA), followed by a post hoc Newman–Keuls comparison multiple test. The level of significance was set at *p* < 0.05.

## 3. Results and Discussion

### 3.1. Phytochemical Profile

Plants possess an abundance of natural compounds also known as secondary metabolites. Plant secondary metabolites are expressed in hundreds of thousands of combinations in different classes of plants, in their different plant parts, at different maturity stages, and under different environmental conditions [20]. Secondary metabolites have been classified into different categories depending on their molecular structure and this study attempted to screen the *B. abyssinica* stem bark extracts for phenols, flavonoids, phenolic acids, flavanols, tannins, and saponins. The main classes of secondary metabolites identified from *B. abyssinica* stem bark extracts were phenols, flavonoids, and saponins. The water extract possessed the highest concentration of phenolic (230.83 mg GAE/g extract), while the methanol extract was rich in flavonoid (75.82 mg RE/g extract), and the ethyl acetate extract possessed the highest amount of saponin (97.37 mg QE/g). As shown in Table 1, the ethyl acetate extract possessed the highest concentration of flavanol and tannin, whereas water and methanol extracts displayed identical amounts of flavanol and tannin. The water extract was rich in phenolic acid. These results were also confirmed by independent HPLC-fluorimetric assays, displaying higher gallic acid and rutin levels in water and ethyl acetate extract, respectively. The HPLC analyses also confirmed identical amounts of rutin in water and methanol extracts, in agreement with colorimetric assays. The results of HPLC analyses are presented in Table 2. Phenols and flavonoids were reported to be the main classes of secondary metabolites responsible for antioxidant properties of plant extracts. In this study, multiple antioxidant assays were conducted to evaluate the antioxidant properties of *B. abyssinica* stem bark extracts and the results are summarized in Table 3. The total antioxidant capacity was estimated using the phosphomolybdenum assay which measures the reduction of Mo (VI) to Mo (V). The results presented in Table 2 demonstrate that the antioxidant capacity of *B. abyssinica* stem bark extracts follow the order water extract > methanol extract > ethyl acetate extract. The reducing potential of the extracts was further determined using the FRAP and CUPRAC methods. The methanol extract (1464.08 mg TE/g extract) showed highest activity in the CUPRAC assay, whereas for FRAP the highest activity was noted for the water extract (1063.70 mg TE/g extract). Radical scavenging assays, namely DPPH and ABTS, revealed that the methanol extract, followed by the water extract showed higher activity as compared with the ethyl acetate extract. The metal chelating evaluation showed that the water extract was the most active. With reference to a previously published paper, *B. abyssinica* stem bark (Table 1) assessed in this study possessed a higher level of phenolic as compared with *B. abyssinica* leaves (36.99, 175.95, and 180.62 mg GAE/g extract for ethyl acetate, methanol, and water extracts, respectively) [21].

In addition to spectrophotometric determination of secondary metabolites, the detailed profiles were elucidated using HPLC-fluorimetric technique. The detailed secondary metabolites profiling provide a better understanding of the secondary metabolites occurrence in herbal extracts and can be used as support for further interaction and mechanisms studies. HPLC-fluorimetric profiling confirmed the presence of 58, 68, and 63 compounds in the ethyl acetate, methanol, and water extracts of *B. abyssinica* stem bark (Table 4, Table 5, and Table 6, respectively). Mangiferin, exhibiting fragment ions at *m*/*z* 343.0470, 331.0464, 301.0358, 272.0331, and 259.0250, was identified in all three extracts. Bruguierol A which exhibited the precursor at *m*/*z* 191.1072 ([M−H]^−^) was identified only in the ethyl acetate and methanol extracts. The flavone, vitexin and its isomer, isovitexin, both exhibiting precursor at *m*/*z* 433.1135 were identified in all the studied extracts. Some compounds could not be identified, for example, a tannin (C_34_H_26_O_2_) exhibiting fragment ions at *m*/*z* 649.0660, 561.0911, 499.0735, 347.0622, and 300.9995; a saponin (C_48_H_76_O_19_) exhibiting fragments ions at *m*/*z* 893.4806, 793.4410, 731.4385, 551.3740, and 455.3530. Kynurenic acid (KA) (*m*/*z* 190,05042 [M−H]^+^) tentatively identified in the methanol and water extracts, has previously been reported to act as a neuromodulator by interacting with nicotinic and GPR35 receptors and regulated the release of neurotransmitters, such as, acetylcholine [22]. The biological activity of *B. abyssinica* stem bark could be related to an individual secondary metabolite or the synergistic action of several compounds.

Enzymes act as catalyst in cellular reactions, and therefore are ideal drug targets [23]. Enzyme inhibitors have been developed to manage several diseases, including Alzheimer’s disease, diabetes type II, and skin hyperpigmentation conditions. The search for a novel candidate stems from the side effects associated with currently used drugs. In the case of Alzheimer’s disease, inhibitors act on cholinesterase enzymes, namely AChE and BChE, which hydrolyze acetylcholine, a neurotransmitter, thereby ending synaptic transmission. Galantamine, rivastigmine, and donepezil are FDA approved drugs used for the management of Alzheimer’s disease. However, these agents have been associated with a number of side effects, such as, vomiting, nausea, muscle cramps, and loss of appetite, thereby advocating the need for novel agents. In this study, *B. abyssinica* stem bark extracts showed inhibitory action against AChE. In addition, the results presented in Table 7 showed that higher galantamine equivalents were recorded against AChE (3.15 to a4.43 mg GALAE/g extract) as compared with BChE (1.27 mg GALAE/g extract), implying that the extracts were more active against AChE as compared with BChE. This study also investigated the possible inhibitory action of *B. abyssinica* stem bark extracts on tyrosinase. Tyrosinase is a rate-limiting enzyme responsible for the biosynthesis of melanin which protects the body against harmful UV rays. However, excessive production of melanin causes hyperpigmentation-related conditions, such as melasma and freckles. Therefore, controlling tyrosinase activity using inhibitors has been revealed to be an ideal therapeutic strategy for the management of skin hyperpigmentation conditions. Here, it was found that *B. abyssinica* stem bark extracts inhibited tyrosinase. The methanol extract exhibited higher inhibition against tyrosinase with a value of 136.51 mg KAE/g extract. However, *B. abyssinica* leaves methanol extract expressed higher inhibitory activity against tyrosinase with a value of 148.94 mg KAE/g extract. Loliolide, monoterpenoid hydroxylactone previously reported to possess anti-melanogenic effects on skin, and other secondary metabolites could be responsible for the observed anti-tyrosinase activity. 

The ability of *B. abyssinica* stem bark extracts to inhibit α-amylase and α-glucosidase was determined. These two carbohydrate hydrolyzing enzymes have been targeted for controlling post-prandial glucose peaks as their activity is directly related to the release of glucose from ingested food. Therefore, inhibition of α-amylase and α-glucosidase blunt glucose rise, thereby preventing hyperglycemia. On the one hand, the results from this study showed that *B. abyssinica* stem bark extracts possessed low inhibition against α-amylase, with acarbose equivalent values per gram of extract ranging from 0.97 to 0.21. On the other hand, the ethyl acetate extract (15.22 mmol ACAE/g extract) showed potent activity against α-glucosidase. It is noteworthy that *B. abyssinica* stem bark ethyl acetate extract was more active than the ethyl acetate extract of *B. abyssinica* leaves (1.09 mmol ACAE/g extract) [21].

### 3.2. Pharmacological Study

**Figure 1:**
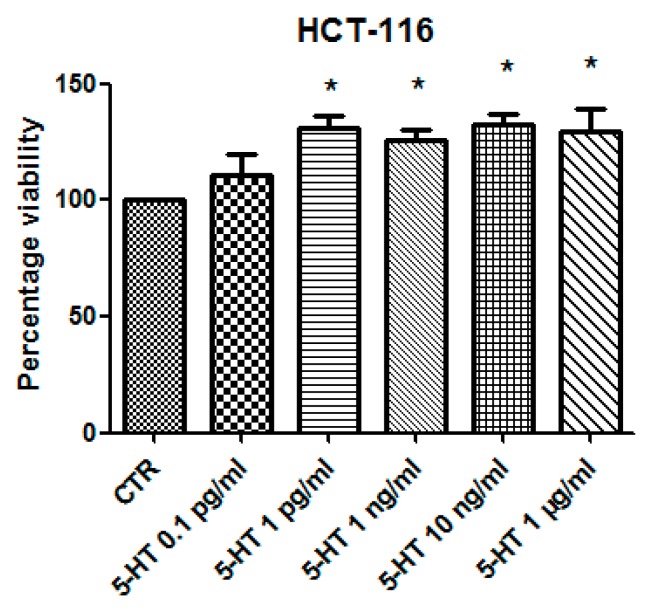
Effects of serotonin (5-HT) on colon cancer HCT116 cell viability (MTT test). Data are means ± SD and analyzed through analysis of variance (ANOVA), followed by post hoc Newman–Keuls test. ANOVA, *p* < 0.01; post hoc, * *p* < 0.05 vs. CTR (control) group.

**Figure 2:**
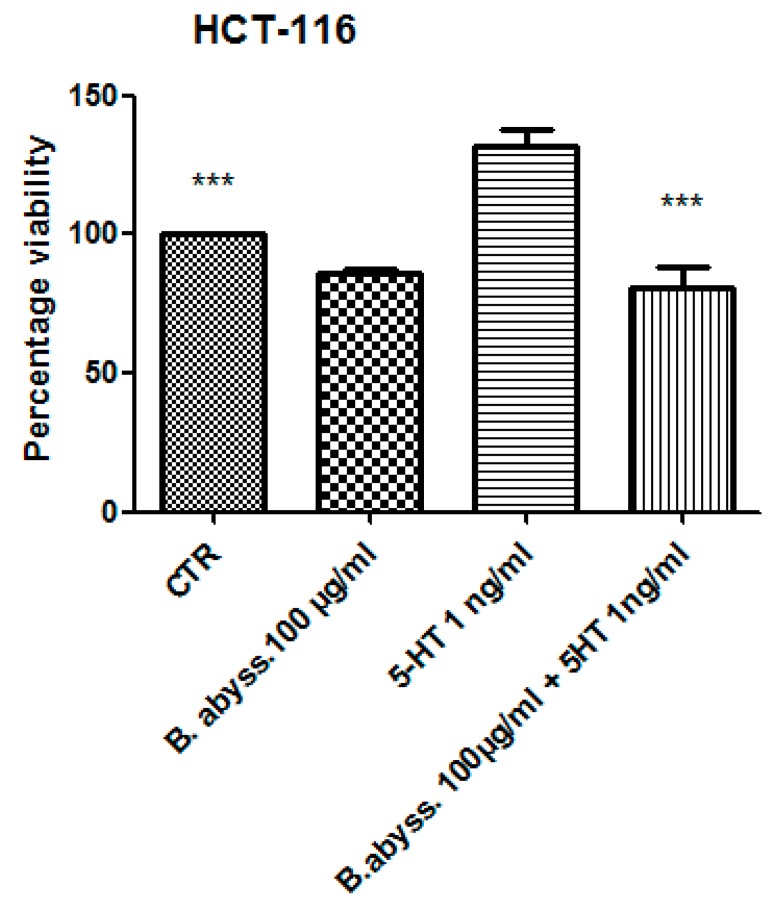
Effects of *B. abyssinica* water extract 100 µg/mL basal and serotonin (5-HT)-induced on colon cancer HCT116 cell viability (MTT test). Data are means ± SD and analyzed through analysis of variance (ANOVA), followed by post hoc Newman–Keuls test. ANOVA, *p* < 0.0001; post hoc, *** *p* < 0.001 vs. 5-HT (serotonin) group. The *B. abyss.* 100 µg/mL group reported in the picture was compared with the sole CTR group (*p* > 0.05).

**Figure 3:**
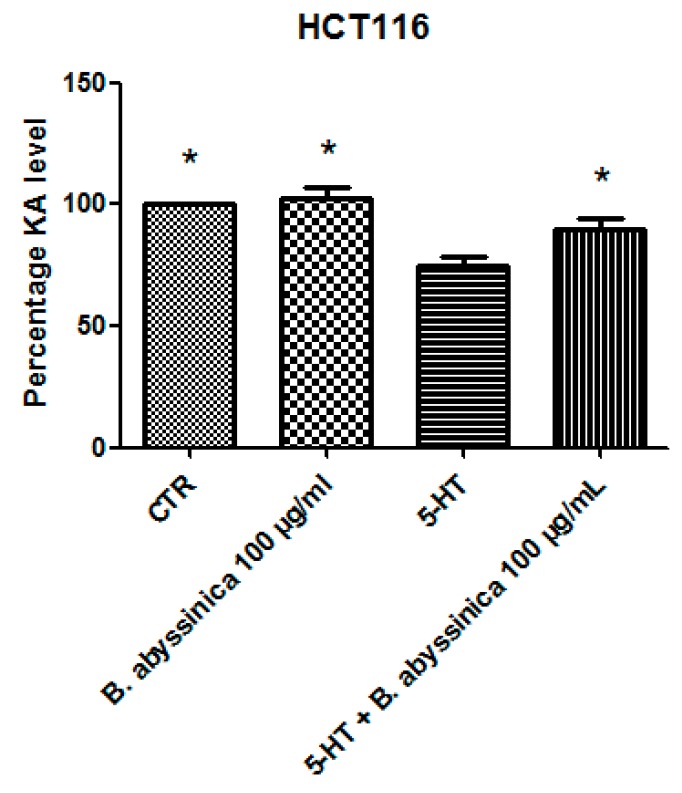
Blunting effect induced by *B. abyssinica* water extract 100 µg/mL on serotonin (5-HT)-induced reduction of kynurenic acid (KA) release from colon cancer HCT116 cells. Data are means ± SD and analyzed through analysis of variance (ANOVA), followed by post hoc Newman–Keuls test. ANOVA, *p* < 0.01; post hoc, * *p* < 0.05 vs. 5-HT (serotonin) group. The *B. abyss.* 100 µg/mL group reported in the picture was compared to the sole CTR group (*p* > 0.05).

**Figure 4:**
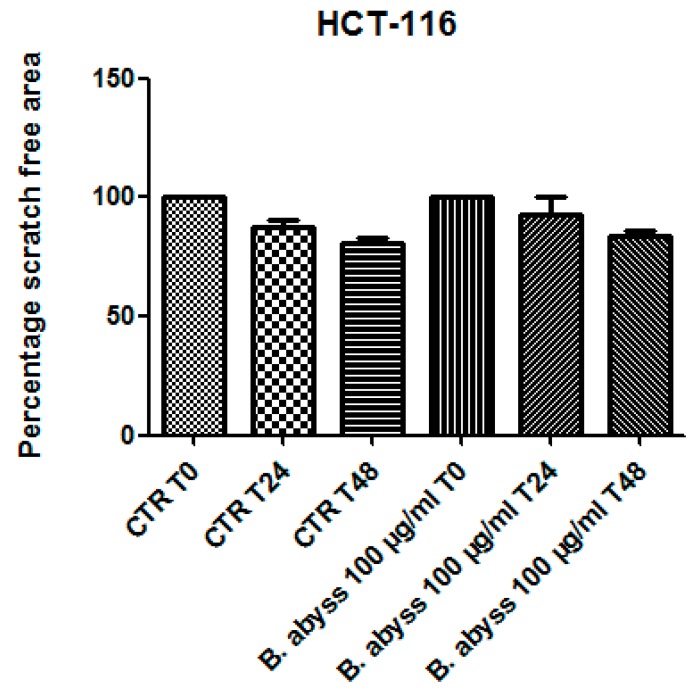
Effects of *B. abyssinica* water extract 100 µg/mL on the spontaneous migration of colon cancer HCT116 cells (wound healing test). The spontaneous migrations were assessed at 0, 24, and 48 h following experimental lesion of the cell monolayer. Data are means ± SD and analyzed through analysis of variance (ANOVA), followed by post hoc Newman–Keuls test. The statistical analysis showed a null effect exerted by the extract on HCT116 cell migration.

Considering the colorimetric and HPLC analysis results, the water extract of *B. abyssinica* which was selected for further investigation aimed to verify the traditional antitumoral use of the plant. Firstly, the biocompatibility limit was determined through the *A. salina* brine shrimp lethality test. The nauplii were treated with *B. abyssinica* water extract, in the range 0.1–20 mg/mL. The lethality test showed a LC_50_ value >1 mg/mL. On the basis of our previous investigation [19], a ten-fold lower concentration (100 µg/mL) was selected for the subsequent in vitro tests. In this regard, the human colon cancer HCT116 cell line was selected and treated with the extract. The effect of water extract was evaluated on both basal and 5-HT-induced cell viability; 5-HT has long been described as a proinflammatory factor, particularly in the gut [10], with in vitro studies substantiating a mitogen role, mediated by different receptor types towards multiple cell lines [9]. According to these findings, a preliminary study was carried out in order to optimize the experimental conditions that could demonstrate a cell viability-stimulating effect of 5-HT, in a wide range of concentrations (0.1 pg/mL to 1 µg/mL). We observed that HCT116 cell viability increased in a concentration-dependent manner, in the range 0.1–1 pg/mL, although it remained constant, at the upper tested concentrations (Figure 1). Considering that our previous ex vivo and in vitro studies focused on inflamed colon specimens and hypothalamic cells, respectively, and reported 5-HT concentrations in the order of ng/mL [16,19], we chose the 5-HT concentration 1 ng/mL as a reliable proliferative stimulus for the following tests. Specifically, the water extract was able to inhibit 5-HT-stimulated viability of HCT116 cells (Figure 2), thus, substantiating the potential antiproliferative effect of the extract in the real in vivo colon cancer cell microenvironment, characterized by the upregulated production of multiple proinflammatory and anti-apoptotic/mitogen factors, including 5-HT [24,25]. This result supports the traditional antitumoral use of *B. abyssinica* [4]. Additionally, in the same experimental conditions, the effects of the extract were evaluated on the extracellular level of kynurenic acid, one of the two main kynurenine metabolites. Kynurenic acid was reportedly produced in multiple tissues, including brain and peripheral organs [26], although pharmacokinetic studies excluded any possibility of the peripheral pool crossing the blood-brain barrier [27]. In the brain, the kynurenine-derived kynurenic acid was described as a reliable marker of neuroprotection [28,29], whereas it seemed to be involved in an inflammatory response at the peripheral level [30]. Kynurenic acid was also described as an antiproliferative factor toward colon, renal, and glioma cells [31]. Specifically, this marker was considered to be a potential chemopreventive agent against colon cancer [31,32], and our findings of reduced kynurenic acid extracellular level, after challenging HCT116 cells with 5-HT, further supports this function. Consistent with its antiproliferative effects, *B. abyssinica* water extract blunted the 5-HT-induced downregulation of kynurenic acid (Figure 3). It is also noteworthy to highlight that kynurenic acid was tentatively identified in the extracts itself through HPLC-MS analysis (Table 6). Nevertheless, the lack of any statistically significant difference in kynurenic acid levels assayed through HPLC-fluorimeter in HCT116 cell groups treated with either vehicle (CTR) or *B. abyssinica* extract (100 µg/mL) (Figure 3), suggested that kynurenic acid concentration in the water extract is much lower as compared with the cell-deriving kynurenic acid pool. Considering the reported inhibition of glioma cell migration induced by kynurenic acid [31], *B. abissynica* water extract was also tested in the wound healing experimental paradigm. The null effect observed on spontaneous cell migration (Figure 4) ruled out any involvement of the extract on invasion capacities of colon cancer cells in vivo. 

## 4. Conclusions

Oxidative stress triggers the onset of several pathologies, but key enzymes remain the main targets in the management of Alzheimer’s disease, diabetes type II, and skin hyperpigmentation problems. Developing agents possessing both antioxidant and enzyme inhibitory properties could be ideal for the holistic management of these complications. Scientific data generated in this work provide baseline data regarding the enzyme inhibitory and antioxidant properties of *B. abyssinica* stem bark, highlighting its potential use for the development of new pharmaceutic and cosmetic agents. In addition, *B. abyssinica* stem bark is regarded as a source of active phytochemical compounds which could be isolated and incorporated in drug formulation for the management of Alzheimer’s disease, diabetes type II, and skin hyperpigmentation complications. Finally, this pharmacological study suggests antiproliferative effects of the *B. abyssinica* water extract that corroborates the traditional use and supports further studies for the confirmation of the antitumoral role in selected colon cancer animal models. 

## Figures and Tables

**Table 1:** Total bioactive components of the tested samples.

Extracts	Total Phenolic Content (mg GAE/g Extract)	Total Flavonoid Content (mg RE/g Extract)	Total Phenolic Acid Content (mg CAE/g)	Total Flavanol Content (mg CE/g)	Total Tannin Content (mg CE/g)	Total Saponin Content (mg QE/g)
EA	100.57 ± 0.67 ^c^	56.25 ± 0.52 ^b^	nd	2.79 ± 0.01 ^a^	2.67 ± 0.16 ^a^	97.37 ± 10.22 ^a^
MeOH	216.79 ± 1.11 ^b^	75.82 ± 0.50 ^a^	2.90 ± 0.46 ^a^	1.23 ± 0.02 ^b^	1.25 ± 0.02 ^b^	76.44 ± 10.70 ^a^
Water	230.83 ± 1.64 ^a^	40.32 ± 0.26 ^c^	3.46 ± 0.04 ^a^	1.23 ± 0.01 ^b^	1.12 ± 0.19 ^b^	57.59 ± 7.44 ^b^

Values expressed are means ± S.D. of three parallel measurements. GAE, gallic acid equivalent; RE, rutin equivalent; CE, catechin equivalent; CAE, caffeic acid equivalent; QE, Quillaja equivalent; EA, ethyl acetate; MeOH, methanol; nd, not detected. Different letters indicate significant differences in the extracts (*p* < 0.05). Superscript letters (a,b,c) indicate different levels of statistical significance.

**Table 2:** Gallic acid and rutin levels in *B. abyssinica* extracts.

Extracts	Gallic Acid (mg/g Extract)	Rutin (mg/g Extract)
EA	1.63 ± 0.15	0.60 ± 0.07
MeOH	3.64 ± 0.33	0.14 ± 0.02
Water	3.26 ± 0.13	0.12 ± 0.01

**Table 3:** Antioxidant activities of the tested samples.

Extracts	DPPH (mg TE/g Extract)	ABTS (mg TE/g Extract)	CUPRAC (mgTE/g Extract)	FRAP (mgTE/g Extract)	Phosphomolybdenum (mmol TE/g)	Metal Chelating Ability (mg EDTAE/g)
EA	392.04 ± 1.83 ^b^	232.90 ± 5.46 ^b^	508.51 ± 2.04 ^b^	363.82 ± 2.00 ^c^	2.48 ± 0.14 ^c^	30.68 ± 1.43 ^b^
MeOH	1092.46 ± 7.38 ^a^	698.86 ± 32.24 ^a^	1464.08 ± 7.46 ^a^	983.86 ± 7.86 ^b^	2.90 ± 0.09 ^b^	32.07 ± 0.55 ^b^
Water	1073.20 ± 22.99 ^a^	560.32 ± 28.57 ^a^	1449.46 ± 6.15 ^a^	1063.70 ± 16.20 ^a^	3.18 ± 0.07 ^a^	49.74 ± 0.11 ^a^

Values expressed are means ± S.D. of three parallel measurements. TE, Trolox equivalent; EDTAE, EDTA equivalent; EA, ethyl acetate; MeOH, methanol. Different letters indicate significant differences in the extracts (*p* < 0.05). Superscript letters (a,b,c) indicate different levels of statistical significance.

**Table 4:** Chemical composition of ethyl acetate extract.

No.	Name	Formula	Rt	[M+H]^+^	[M−H]^−^	Fragment 1	Fragment 2	Fragment 3	Fragment 4	Fragment 5
1	Galloylhexose isomer 1	C_13_H_16_O_10_	1.26		331.07	271.05	241.03	211.02	169.01	125.02
2	Citric acid	C_6_H_8_O_7_	1.56		191.02	173.01	129.02	111.01	87.01	85.03
3	Galloylhexose isomer 2	C_13_H_16_O_10_	1.70		331.07	271.05	241.04	211.02	169.01	125.02
4	Galloylhexose isomer 3	C_13_H_16_O_10_	2.19		331.07	271.05	241.03	211.02	169.01	125.02
5 ^1^	Gallic acid (3,4,5-trihydroxybenzoic acid)	C_7_H_6_O_5_	2.30		169.01	125.02	97.03	81.03	79.02	69.03
6	Galloylhexose isomer 4	C13H16O10	2.82		331.07	271.05	241.04	211.02	169.01	125.02
7	Protocatechuic acid (3,4-dihydroxybenzoic acid)	C7H6O4	4.65		153.02	109.03	108.02	91.02	81.03	
8	Syringic acid-O-hexoside isomer 1	C15H20O10	10.17		359.10	197.05	182.02	167.00	153.05	138.03
9	Syringic acid-O-hexoside isomer 2	C15H20O10	10.60		359.10	197.05	182.02	167.00	153.05	138.03
10	Caffeic acid	C9H8O4	14.28		179.03	135.04	107.05			
11	Unidentified tannin	C34H26O23	14.38		801.08	649.07	561.09	499.07	347.06	301.00
12	Trigalloylhexose isomer 1	C27H24O18	14.46		635.09	483.08	465.07	313.06	169.01	125.02
13	Digalloylhexose	C20H20O14	15.18		483.08	331.07	313.06	271.05	169.01	125.02
14	Trigalloylhexose isomer 2	C27H24O18	15.37		635.09	483.08	465.07	313.06	169.01	125.02
15	Trigalloylhexose isomer 3	C27H24O18	16.56		635.09	483.07	465.07	313.06	169.01	125.02
16	Ethyl syringate	C11H14O5	16.68	227.09		181.05	155.07	140.05	123.04	95.05
17	Feruloylhexose isomer 1	C16H20O9	16.73		355.10	295.08	235.06	193.05	175.04	134.04
18	Trigalloylhexose isomer 4	C27H24O18	17.31		635.09	483.08	465.07	313.06	169.01	125.02
19	Synapoylhexose isomer 1	C17H22O10	17.56		385.11	325.09	265.07	223.06	205.05	190.03
20^1^	4-Coumaric acid	C9H8O3	17.70		163.04	119.05	93.03			
21	Feruloylhexose isomer 2	C16H20O9	17.84		355.10	295.08	235.06	193.05	175.04	134.04
22	Myrciaphenone B	C21H22O13	17.87		481.10	313.06	169.01	125.02		
23	Mangiferin (Aphloiol, Chinonin)	C19H18O11	18.38		421.08	343.05	331.05	301.04	272.03	259.03
24	Synapoylhexose isomer 2	C17H22O10	18.53		385.11	325.09	265.07	223.06	205.05	190.03
25	Ferulic acid	C10H10O4	19.24		193.05	178.03	149.06	137.02	134.04	
26	Tetragalloylhexose	C34H28O22	19.36		787.10	635.09	617.08	465.07	313.06	169.01
27	Loliolide	C11H16O3	19.45	197.12		179.11	161.10	135.12	133.10	107.09
28	Ellagic acid-4-O-glucoside	C20H16O13	19.89		463.05	301.00	299.99	298.98		
29	Feruloyl-galloylhexose	C23H24O13	20.98		507.11	313.06	193.05	179.03	169.01	125.02
30	Quercetin-O-galloylhexoside isomer 1	C28H24O16	21.31		615.10	463.09	301.03	300.03	255.03	169.01
31^1^	Vitexin (Apigenin-8-C-glucoside)	C21H20O10	21.34	433.11		415.10	397.09	367.08	313.07	283.06
32	Quercetin-O-galloylhexoside isomer 2	C28H24O16	21.56		615.10	463.09	301.04	300.03	271.02	169.01
33	Isovitexin (Apigenin-6-C-glucoside)	C21H20O10	22.24	433.11		415.10	397.09	367.08	313.07	283.06
34	Sinapoyl-galloylhexose	C24H26O14	22.36		537.12	325.09	265.07	223.06	169.01	125.02
35	Hyperoside (Quercetin-3-O-galactoside)	C21H20O12	22.64		463.09	301.04	300.03	271.02	255.03	151.00
36	Ellagic acid-O-pentoside	C19H14O12	22.75		433.04	301.00	299.99	298.99		
37	Acetylmangiferin	C21H20O12	23.11		463.09	331.05	301.04	271.03	259.03	
38	Ellagic acid	C14H6O8	23.37		301.00	284.00	257.01	229.01	201.02	185.02
39	Avicularin (Quercetin-3-O-arabinofuranoside)	C20H18O11	23.45		433.08	301.04	300.03	271.03	255.03	179.00
40	Unidentified terpenoid	C26H34O8	24.67	475.23		379.19	361.18	351.20	333.19	315.18
41	Isorhamnetin-O-hexoside	C22H22O12	24.70		477.10	315.05	314.04	285.04	271.03	243.03
42	Kaempferol-O-pentoside	C20H18O10	24.83		417.08	285.04	284.03	255.03	227.03	
43^1^	Isorhamnetin-3-O-glucoside	C22H22O12	24.88		477.10	315.05	314.04	285.04	271.03	243.03
44	4-Methoxycinnamic acid	C10H10O3	25.31	179.07		161.06	133.07	105.07	79.05	
45	3-O-Methylellagic acid	C15H8O8	25.66		315.01	299.99	244.00			
46	Tetrahydroxyxanthone	C13H8O6	26.12		259.02	231.03	215.03	203.03	187.04	
47	Dihydroactinidiolide	C11H16O2	26.57	181.12		163.11	145.10	135.12	107.09	93.07
48^1^	Naringenin (4’,5,7-trihydroxyflavanone)	C15H12O5	27.15		271.06	177.02	151.00	119.05	107.01	93.03
49	3,3’-Di-O-methylellagic acid	C16H10O8	27.81		329.03	314.01	298.98	270.99		
50	3,3’,4-Tri-O-methylellagic acid	C17H12O8	30.17		343.05	328.02	313.00	297.98	285.00	
51	3,3’,4-Tri-O-methylflavellagic acid	C17H12O9	31.21		359.04	344.02	328.99	313.97	301.00	
52	3,3’,4,4’-Tetra-O-methylellagic acid	C18H14O8	31.98	359.08		344.05	343.05	329.03	313.03	
53	Bruguierol A	C12H14O2	36.05	191.11		173.10	161.10	147.08	135.08	107.05
54	Ginsenoside Ro or isomer	C48H76O19	36.23		955.49	793.44	731.44	613.37	569.38	551.38
55	Cynarasaponin C or isomer	C42H66O14	37.20		793.44	631.39	587.40	569.38	497.37	455.35
56	Ginsenoside Ro or isomer	C48H76O19	39.75		955.49	793.44	731.44	569.38	551.37	455.35
57	Unidentified saponin	C48H76O19	40.25		955.49	893.48	793.44	731.44	551.37	455.35
58	Hexadecanedioic acid	C16H30O4	40.30		285.21	267.20	223.21	57.03		

^1^ Confirmed by standard.

**Table 5:** Chemical composition of methanol extract.

No.	Name	Formula	Rt	[M+H]^+^	[M−H]^−^	Fragment 1	Fragment 2	Fragment 3	Fragment 4	Fragment 5
1	Galloylhexose isomer 1	C13H16O10	1.22		331.07	271.05	241.03	211.02	169.01	125.02
2	Citric acid	C6H8O7	1.56		191.02	173.01	129.02	111.01	87.01	85.03
3	Galloylhexose isomer 2	C13H16O10	1.71		331.07	271.05	241.04	211.02	169.01	125.02
4	Galloylhexose isomer 3	C13H16O10	2.18		331.07	271.05	241.03	211.02	169.01	125.02
5 ^1^	Gallic acid (3,4,5-trihydroxybenzoic acid)	C7H6O5	2.31		169.01	125.02	97.03	81.03	79.02	69.03
6	Galloylhexose isomer 4	C13H16O10	2.81		331.07	271.05	241.03	211.02	169.01	125.02
7	Protocatechuic acid (3,4-dihydroxybenzoic acid)	C7H6O4	4.65		153.02	109.03	108.02	91.02	81.03	
8	Syringic acid-O-hexoside isomer 1	C15H20O10	10.19		359.10	197.05	182.02	167.00	153.05	138.03
9	Syringic acid-O-hexoside isomer 2	C15H20O10	10.61		359.10	197.05	182.02	167.00	153.05	138.03
10	Kynurenic acid	C10H7NO3	13.07	190.05		162.06	144.04	116.05		
11	Caffeic acid	C9H8O4	14.28		179.03	135.04	107.05			
12	Unidentified tannin	C34H26O23	14.37		801.08	649.07	561.09	499.07	347.06	301.00
13	Trigalloylhexose isomer 1	C27H24O18	14.46		635.09	483.08	465.07	313.06	169.01	125.02
14	Digalloylhexose	C20H20O14	15.19		483.08	331.07	313.06	271.05	169.01	125.02
15	Trigalloylhexose isomer 2	C27H24O18	15.37		635.09	483.08	465.07	313.06	169.01	125.02
16	Castalin or vescalin	C27H20O18	15.65		631.06	451.00	301.00	299.99	298.98	270.99
17	Methylcoumarin	C10H8O2	15.93	161.06		133.07	105.07	91.05		
18	Castalin or vescalin	C27H20O18	16.38		631.06	451.00	301.00	299.99	298.98	270.99
19	Trigalloylhexose isomer 3	C27H24O18	16.57		635.09	483.08	465.07	313.06	169.01	125.02
20	Ethyl syringate	C11H14O5	16.67	227.09		181.05	155.07	140.05	123.04	95.05
21	Feruloylhexose isomer 1	C16H20O9	16.73		355.10	295.08	235.06	193.05	175.04	134.04
22	Trigalloylhexose isomer 4	C27H24O18	17.31		635.09	483.08	465.07	313.06	169.01	125.02
23	Synapoylhexose isomer 1	C17H22O10	17.56		385.11	325.09	265.07	223.06	205.05	190.03
24^1^	4-Coumaric acid	C9H8O3	17.70		163.04	119.05	93.03			
25	Feruloylhexose isomer 2	C16H20O9	17.84		355.10	295.08	235.06	193.05	175.04	134.04
26	Myrciaphenone B	C21H22O13	17.86		481.10	313.06	169.01	125.02		
27	Mangiferin (Aphloiol, Chinonin)	C19H18O11	18.33		421.08	343.05	331.05	301.04	272.03	259.02
28	Synapoylhexose isomer 2	C17H22O10	18.52		385.11	325.09	265.07	223.06	205.05	190.03
29	Ferulic acid	C10H10O4	19.24		193.05	178.03	149.06	137.02	134.04	
30	Loliolide	C11H16O3	19.46	197.12		179.11	161.10	135.12	133.10	107.09
31	Tetragalloylhexose	C34H28O22	19.47		787.10	635.09	617.08	465.07	313.06	169.01
32	Ellagic acid-4-O-glucoside	C20H16O13	19.90		463.05	301.00	299.99	298.98		
33	Feruloyl-galloylhexose	C23H24O13	20.99		507.11	313.06	193.05	179.03	169.01	125.02
34	Berscillogenin or 3-epiberscillogenin	C24H30O6	21.30	415.21		397.20	379.19	367.19	361.18	351.20
35	Quercetin-O-galloylhexoside isomer 1	C28H24O16	21.33		615.10	463.09	301.04	300.03	255.03	169.01
36^1^	Vitexin (Apigenin-8-C-glucoside)	C21H20O10	21.37	433.11		415.10	397.09	367.08	313.07	283.06
37	Quercetin-O-galloylhexoside isomer 2	C28H24O16	21.55		615.10	463.09	301.04	300.03	271.02	169.01
38	Berscillogenin or 3-epiberscillogenin	C24H30O6	22.02	415.21		397.21	379.19	367.19	361.18	351.19
39	Isovitexin (Apigenin-6-C-glucoside)	C21H20O10	22.24	433.11		415.10	397.09	367.08	313.07	283.06
40	Sinapoyl-galloylhexose	C24H26O14	22.37		537.12	325.09	265.07	223.06	169.01	125.02
41	Hyperoside (Quercetin-3-O-galactoside)	C21H20O12	22.65		463.09	301.04	300.03	271.03	255.03	151.00
42	Ellagic acid-O-pentoside	C19H14O12	22.75		433.04	301.00	299.99	298.99		
43^1^	Isoquercitrin (Quercetin-3-O-glucoside)	C21H20O12	22.89		463.09	301.04	300.03	271.03	255.03	151.00
44	Acetylmangiferin	C21H20O12	23.11		463.09	331.05	301.04	271.02	259.02	
45	Eschweilenol A	C20H10O11	23.27		425.01	301.00	299.99	298.98		
46	Ellagic acid	C14H6O8	23.35		301.00	284.00	257.01	229.01	201.02	185.02
47	Avicularin (Quercetin-3-O-arabinofuranoside)	C20H18O11	23.47		433.08	301.04	300.03	271.03	255.03	179.00
48	Unidentified terpenoid	C26H34O8	24.66	475.23		379.19	361.18	351.20	333.19	315.17
49	Isorhamnetin-O-hexoside	C22H22O12	24.70		477.10	315.05	314.04	285.04	271.02	243.03
50	Kaempferol-O-pentoside	C20H18O10	24.84		417.08	285.04	284.03	255.03	227.03	
51^1^	Isorhamnetin-3-O-glucoside	C22H22O12	24.90		477.10	315.05	314.04	285.04	271.03	243.03
52	4-Methoxycinnamic acid	C10H10O3	25.32	179.07		161.06	133.07	105.07	79.05	
53	3-O-Methylellagic acid	C15H8O8	25.66		315.01	299.99	244.00			
54	Tetrahydroxyxanthone	C13H8O6	26.13		259.02	231.03	215.03	203.03	187.04	
55	Dihydroactinidiolide	C11H16O2	26.58	181.12		163.11	145.10	135.12	107.09	93.07
56^1^	Naringenin (4’,5,7-trihydroxyflavanone)	C15H12O5	27.17		271.06	177.02	151.00	119.05	107.01	93.03
57	3,3’-Di-O-methylellagic acid	C16H10O8	27.82		329.03	314.01	298.98	270.99		
58	Scilliglaucosidin	C24H30O5	27.87	399.22		381.21	363.19	345.19	335.20	
59	3,3’,4-Tri-O-methylellagic acid	C17H12O8	30.18		343.05	328.02	313.00	297.98	285.00	
60	3,3’,4-Tri-O-methylflavellagic acid	C17H12O9	31.21		359.04	344.02	328.99	313.97	301.00	
61	3,3’,4,4’-Tetra-O-methylellagic acid	C18H14O8	31.98	359.08		344.05	343.05	329.03	313.03	
62	Bruguierol A	C12H14O2	36.05	191.11		173.10	161.10	147.08	135.08	107.05
63	Ginsenoside Ro or isomer	C48H76O19	36.24		955.49	793.44	731.44	613.37	569.38	551.37
64	Cynarasaponin C or isomer	C42H66O14	37.18		793.44	631.39	587.40	569.39	497.37	455.35
65	Ginsenoside Ro or isomer	C48H76O19	39.71		955.49	793.44	731.44	569.38	551.37	455.35
66	Unidentified saponin 1	C48H76O19	40.28		955.49	893.49	793.44	731.44	551.37	455.35
67	Hexadecanedioic acid	C16H30O4	40.29		285.21	267.20	223.21	57.03		
68	Unidentified saponin 2	C49H78O19	41.70		969.51	951.50	585.38	537.36	455.35	453.33

^1^ Confirmed by standard.

**Table 6:** Chemical composition of water extract.

No.	Name	Formula	Rt	[M+H]^+^	[M−H]^−^	Fragment 1	Fragment 2	Fragment 3	Fragment 4	Fragment 5
1	Galloylhexose isomer 1	C13H16O10	1.31		331.07	271.05	241.04	211.02	169.01	125.02
2	Citric acid	C6H8O7	1.55		191.02	173.01	129.02	111.01	87.01	85.03
3	Galloylhexose isomer 2	C13H16O10	1.76		331.07	271.05	241.03	211.02	169.01	125.02
4	Galloylhexose isomer 3	C13H16O10	2.12		331.07	271.05	241.03	211.02	169.01	125.02
5 ^1^	Gallic acid (3,4,5-trihydroxybenzoic acid)	C7H6O5	2.28		169.01	125.02	97.03	81.03	79.02	69.03
6	Galloylhexose isomer 4	C13H16O10	2.79		331.07	271.05	241.04	211.02	169.01	125.02
7	Protocatechuic acid (3,4-dihydroxybenzoic acid)	C7H6O4	4.69		153.02	109.03	108.02	91.02	81.03	
8	Piscidic acid (4-hydroxybenzyltartaric acid)	C11H12O7	5.40		255.05	193.05	179.03	165.05	149.06	72.99
9	Syringic acid-O-hexoside	C15H20O10	10.58		359.10	197.05	182.02	167.00	153.05	138.03
10	Kynurenic acid	C10H7NO3	13.07	190.05		162.06	144.04	116.05		
11	Caffeic acid	C9H8O4	14.32		179.03	135.04	107.05			
12	Unidentified tannin	C34H26O23	14.40		801.08	649.07	561.09	499.07	347.06	301.00
13	Trigalloylhexose isomer 1	C27H24O18	14.48		635.09	483.08	465.07	313.06	169.01	125.02
14	Digalloylhexose	C20H20O14	15.21		483.08	331.07	313.06	271.05	169.01	125.02
15	Trigalloylhexose isomer 2	C27H24O18	15.40		635.09	483.08	465.07	313.06	169.01	125.02
16	Castalin or vescalin	C27H20O18	15.69		631.06	450.99	301.00	299.99	298.98	270.99
17	Methylcoumarin	C10H8O2	15.92	161.06		133.07	105.07	91.05		
18	Castalin or vescalin	C27H20O18	16.38		631.06	451.00	301.00	299.98	298.98	270.99
19	Trigalloylhexose isomer 3	C27H24O18	16.57		635.09	483.08	465.07	313.06	169.01	125.02
20	Ethyl syringate	C11H14O5	16.69	227.09		181.05	155.07	140.05	123.04	95.05
21	Feruloylhexose isomer 1	C16H20O9	16.75		355.10	295.08	235.06	193.05	175.04	134.04
22	Trigalloylhexose isomer 4	C27H24O18	17.32		635.09	483.08	465.07	313.06	169.01	125.02
23	Synapoylhexose isomer 1	C17H22O10	17.57		385.11	325.09	265.07	223.06	205.05	190.03
24^1^	4-Coumaric acid	C9H8O3	17.71		163.04	119.05	93.03			
25	Feruloylhexose isomer 2	C16H20O9	17.85		355.10	295.08	235.06	193.05	175.04	134.04
26	Myrciaphenone B	C21H22O13	17.86		481.10	313.06	169.01	125.02		
27	Mangiferin (Aphloiol, Chinonin)	C19H18O11	18.33		421.08	343.05	331.05	301.04	272.03	259.03
28	Synapoylhexose isomer 2	C17H22O10	18.53		385.11	325.09	265.07	223.06	205.05	190.03
29	Loliolide	C11H16O3	19.46	197.12		179.11	161.10	135.12	133.10	107.09
30	Tetragalloylhexose	C34H28O22	19.49		787.10	635.09	617.08	465.07	313.06	169.01
31	Ellagic acid-4-O-glucoside	C20H16O13	19.90		463.05	301.00	299.99	298.98		
32	Feruloyl-galloylhexose	C23H24O13	20.97		507.11	313.06	193.05	179.03	169.01	125.02
33	Quercetin-O-galloylhexoside isomer 1	C28H24O16	21.32		615.10	463.09	301.04	300.03	255.03	169.01
34^1^	Vitexin (Apigenin-8-C-glucoside)	C21H20O10	21.34	433.11		415.10	397.09	367.08	313.07	283.06
35	Quercetin-O-galloylhexoside isomer 2	C28H24O16	21.56		615.10	463.09	301.04	300.03	271.02	169.01
36	Isovitexin (Apigenin-6-C-glucoside)	C21H20O10	22.26	433.11		415.10	397.09	367.08	313.07	283.06
37	Sinapoyl-galloylhexose	C24H26O14	22.37		537.12	325.09	265.07	223.06	169.01	125.02
38	Hyperoside (Quercetin-3-O-galactoside)	C21H20O12	22.64		463.09	301.04	300.03	271.03	255.03	151.00
39	Ellagic acid-O-pentoside	C19H14O12	22.75		433.04	301.00	299.99	298.98		
40^1^	Isoquercitrin (Quercetin-3-O-glucoside)	C21H20O12	22.88		463.09	301.04	300.03	271.03	255.03	151.00
41	Acetylmangiferin	C21H20O12	23.10		463.09	331.05	301.04	271.03	259.03	
42	Ellagic acid	C14H6O8	23.36		301.00	284.00	257.01	229.01	201.02	185.02
43	Avicularin (Quercetin-3-O-arabinofuranoside)	C20H18O11	23.48		433.08	301.04	300.03	271.03	255.03	179.00
44	Unidentified terpenoid	C26H34O8	24.67	475.23		379.19	361.18	351.20	333.19	315.17
45	Isorhamnetin-O-hexoside	C22H22O12	24.70		477.10	315.05	314.04	285.04	271.03	243.03
46	Kaempferol-O-pentoside	C20H18O10	24.85		417.08	285.04	284.03	255.03	227.03	
47^1^	Isorhamnetin-3-O-glucoside	C22H22O12	24.90		477.10	315.05	314.04	285.04	271.03	243.03
48	4-Methoxycinnamic acid	C10H10O3	25.32	179.07		161.06	133.07	105.07	79.05	
49	3-O-Methylellagic acid	C15H8O8	25.66		315.01	299.99	244.00			
50	Tetrahydroxyxanthone	C13H8O6	26.12		259.02	231.03	215.03	203.03	187.04	
51	Dihydroactinidiolide	C11H16O2	26.57	181.12		163.11	145.10	135.12	107.09	93.07
52^1^	Naringenin (4’,5,7-trihydroxyflavanone)	C15H12O5	27.17		271.06	177.02	151.00	119.05	107.01	93.03
53	Di-O-methylellagic acid isomer 1	C16H10O8	27.41		329.03	314.01	298.98	270.99		
54	3,3’-Di-O-methylellagic acid	C16H10O8	27.81		329.03	314.01	298.98	270.99		
55	Scilliglaucosidin	C24H30O5	27.88	399.22		381.21	363.20	345.18	335.20	
56	Di-O-methylellagic acid isomer 2	C16H10O8	28.22		329.03	314.01	298.98	270.99		
57	3,3’,4-Tri-O-methylellagic acid	C17H12O8	30.16		343.05	328.02	313.00	297.98	285.00	
58	3,3’,4,4’-Tetra-O-methylellagic acid	C18H14O8	31.99	359.08		344.05	343.05	329.03	313.04	
59	Ginsenoside Ro or isomer	C48H76O19	36.24		955.49	793.44	731.44	613.37	569.39	551.38
60	Cynarasaponin C or isomer	C42H66O14	37.18		793.44	631.38	587.40	569.39	497.36	455.35
61	Ginsenoside Ro or isomer	C48H76O19	39.73		955.49	793.45	731.44	569.38	551.38	455.35
62	Unidentified saponin	C48H76O19	40.27		955.49	893.48	793.44	731.44	551.38	455.35
63	Hexadecanedioic acid	C16H30O4	40.29		285.21	267.20	223.21	57.03		

^1^ Confirmed by standard.

**Table 7:** Enzyme inhibitory properties of the tested extracts.

Extracts	AChE (mg GALAE/g Extract)	BChE (mg GALAE/g Extract)	Tyrosinase (mg KAE/g Extract)	α-Amylase (mmol ACAE/g Extract)	α-Glucosidase (mmol ACAE/g Extract)
EA	4.43 ± 0.15 ^a^	1.27 ± 0.06	127.36 ± 0.98 ^b^	0.97 ± 0.06 ^a^	15.22 ± 0.11
MeOH	4.00 ± 0.05 ^a^	na	136.51 ± 0.70 ^a^	0.68 ± 0.03 ^b^	na
Water	3.15 ± 0.02 ^b^	na	81.63 ± 2.10 ^c^	0.21 ± 0.01 ^c^	na

Values expressed are means ± S.D. of three parallel measurements. GALAE, galatamine equivalent; KAE, kojic acid equivalent; ACAE, acarbose equivalent; na, not active; EA, ethyl acetate; MeOH, methanol. Different letters indicate significant differences in the extracts (*p* < 0.05). Superscript letters (a,b,c) indicate different levels of statistical significance.

## Supplementary Materials

The following are available online at https://www.mdpi.com/2076-3921/9/2/163/s1, Supplementary Materials and Methods, Figure S1: Total ion chromatograms obtained for methanol extract in positive (a) and negative (b) mode, Figure S2: Total ion chromatograms obtained for ethyl acetate extract in positive (a) and negative (b) mode, Figure S3: Total ion chromatograms obtained for water extract in positive (a) and negative (b) mode
